# The Mechanics of Social Interactions Between Cats and Their Owners

**DOI:** 10.3389/fvets.2021.650143

**Published:** 2021-03-31

**Authors:** Dennis C. Turner

**Affiliations:** ^1^Institute for Applied Ethology and Animal Psychology, I.E.A.P./I.E.T., Horgen, Switzerland; ^2^Vetsuisse Faculty, University of Zurich, Zurich, Switzerland

**Keywords:** owners, socialization, communication, mood, cats, interactions, breed

## Abstract

This is a mini review that summarizes what is known from quantitative observational studies of social interactions between domestic cats and humans in both laboratory colonies and the home setting. Only results from data that have been statistically analyzed are included; hypotheses still to be tested will be declared as such. In some cases, the observational data have been combined with independently collected subjective assessments by the owners of the animals' character and owner personality traits to help interpret the data. Further some relevant experimental studies are also included. All social interactions between cats and humans that are discussed below assume that the animals were socialized to people as kittens, the first topic of this review. Such socialized cats show what might be called “friendliness to humans,” which in turn affects human attachment to the cat. The visual and acoustic behavioral elements used to communicate and interact with other cats can be perceived by people and are also employed by the cats when interacting with them. The initiation, and the initiator of social interactions between cats and humans have been shown to influence both the duration of the interaction bout and total interaction time in the relationship. Compliance with the interactional “wishes” of the partner is positively correlated between the cats and the humans over all human-cat dyads examined. Cats do not spontaneously prefer one gender or age cohort of people, but the humans in those cohorts behave differently to the cats causing the latter to react differentially. The dyadic interaction structure has also been shown to differ between women and men and between older and younger adults. Nevertheless, cats—merely their presence but of course their behavior—can affect human moods and human mood differences have been shown to affect the behavior of the cats. Finally, differences have been found between interactions with purebred and non-purebred cats and between younger and older cats.

## Socialization and Other Factors Affecting Establishment of a New Relationship

Eileen Karsh was the first researcher to experimentally determine the sensitive phase of kittens for socialization to humans and this was supported by further data from cat colonies in Zurich and Cambridge (1–3). Kittens handled frequently by humans during their second to mid-seventh week of age become friendly and trusting of people and remain so throughout their later lives [tested to at least 3 years of age, (4)]. The duration and frequency of handling and number of handlers required for this effect have also been examined (5). Much behavior toward conspecifics is still to be learned. Schaer (6) suggested that conspecific “socialization” occurs by about 10 weeks and Hediger (2) confirmed experimentally that socialization to conspecifics and to humans can occur simultaneously. Therefore, most experts recommend not placing kittens before 10 or 12 weeks of age (7)^1^.

Although original socialization status to people is of paramount importance for future cat-human relationships, other parameters have also been shown to influence the establishment of a new relationship [summarized in a model by (1, 8)]: genes of the father (9); presence and behavior of the mother (10); curiosity (exploratory behavior, see below); stroking the cat; and the act of feeding the animal (11). The model by Turner predicts differential outcomes of later positive and negative experiences with people depending on the quality of original socialization to humans. For a cat well-socialized to humans as a kitten it takes many negative experiences with other people to become wary of such contacts and very few positive experiences with a new owner to become friendly and trusting of that person. A cat poorly socialized to people as a kitten requires a great deal of positive experience to accept a new person, but very little negative experience with a person to confirm its wariness and fear of people. Most shelter employees will inform that a poorly socialized and/or mishandled cat requires a great deal of patience and understanding by the new owner after being rehomed, while a well-socialized individual will take only 1–2 weeks to adapt to the new owner and home. This has enormous welfare implications for the cats involved in that poorly socialized cats take up limited space in the shelter for longer while waiting for the personnel to find such a patient new owner, and well-socialized cats can be rehomed more easily and quickly.

## Friendliness to Humans

Turner et al. (9) reported a father effect on the behavioral patterns of kittens associated with what one might call “friendliness to humans.” Since cat males have nothing to do with raising their kittens, this effect had to be genetic. At the time the authors cautioned that they were not talking about a “gene for friendliness” and later, McCune (3) proposed that the genetic father effect was on “boldness” of his kittens, which in turn, increased or decreased their exploratory behavior and the chances of their contact with new humans, appearing as friendliness or, if lower, shyness.

Turner and Stammbach-Geering (12) asked women living at home to subjectively assess their cats and relationships to them along 31 traits, once for their current cat and once for the “ideal” cat and relationship. The effects of civil status, housing condition (indoor or with outdoor access), and number of cats kept on the trait ratings were also examined.

Significant positive correlations were found between the ratings of “cat affection to the owner” and “owner affection for the cat.” The former was positively correlated with ratings for “predictability,” “proximity to the owner,” “enjoyment of physical contact,” “cleanliness” and “likeness to humans.” The keepers of cats with outdoor access rated their animals as being less curious than those of indoor cats. The authors hypothesized that cats kept exclusively indoors were compensating for their less animate environment by initiating more contacts with objects inside than the outdoor cats did. However, it is important to remember that correlational results are not necessarily causal, and still need to be tested experimentally. Turner's (13) observational data on human contact initiation by indoor cats do however support the hypothesized interpretation.

## Communication Between Cats and With Humans

Cat-cat visual, olfactory and auditory communication have been fairly well deciphered beginning with Leyhausen's (14) original work on the body and facial signals used [expanded by (15–17)]. Cats often use some of the same visual and vocal signals when interacting with people. When they approach another familiar cat and greet their keepers after a short absence, they raise their tails upright, presumably as a sign of friendly intentions. Only domesticated cats use this signal and it has been suggested that there was selective pressure for such a signal in the dense temple colonies of ancient Egypt (18). To get our attention, they flank-rub on our legs (which might also mark us) and head-rub—forehead to forehead—with cats they know well, presumably marking each other (and us) with a scent (1, 19). Bernstein and Friedmann [(20), also citing (21)] reported that cats preferred certain places on their bodies, particularly the head region, for being stroked, modified their postures to promote access to those preferred regions, and even led their keepers to preferred places in the home for petting episodes. Ellis et al. (22) determined that both handler familiarity and body region stroked significantly influenced negative behavioral responses. Bernstein and Friedmann (op cit.) also mentioned the cat's closing of the eyes in this relaxed situation (sometimes called the “slow blink”). This slow-blink has received more attention recently and when previously unfamiliar persons initiate such blinking, cats tend to approach them more often (23).

Auditory communication by cats has been and continues to be examined [reviewed by (17)], most recently by Schötz et al. (24) using phonetic analyses of cat-to-human vocalizations. It is generally known that cats vocalize more frequently with their human companions than with other cats (1). Yeon et al. (25) found that meows are attention-seeking vocalizations in interspecific situations and higher pitched (subjectively more pleasant) than in feral cats and wild ancestors. They also modify their purrs when actively soliciting food (more urgent and less pleasant than when just resting as perceived by the human raters) and people are capable of distinguishing these (26), both behaviors probably learned over time in interactions. Ellis et al. (27) reported that 40% of their human participants identified the correct contexts of cat vocalizations more often than by chance when the vocalizations belonged to their own cat, but did not perform above chance when the calls belonged to an unfamiliar cat. Interestingly, Saito et al. (28) demonstrated with the habituation-dishabituation method that privately owned cats can discriminate their own names from other words, which leads now to other studies in the area of social cognition in cats.

Recent work on social cognition in cats also has relevance to cat-human communication. Vitale Shreve and Udell (29) provided a first review of what was known and still to be discovered and a number of studies have since been published. Pongracz and his colleagues in Hungary have been particularly active this this area. Even though Miklosi et al. (30) had already shown differences between dogs and cats in their ability to use human pointing gestures, especially that cats lacked some components of attention-getting behavior compared with dogs, Pongracz et al. (31) demonstrated that cats were indeed able to read and follow human gaze for referential information. Galvan and Vonk (32) found that cats were only modestly sensitive to emotions as indicated by human postural and vocal cues, but particularly when displayed by their owner. Quaranta et al. (33) demonstrated experimentally that cats are indeed capable to cross-modally match pictures of emotional faces with their related vocalizations in both conspecifics and humans, especially for high intensity emotions. These authors concluded that cats have a general mental representation for the emotions of their social partners, both conspecific and human.

## The Initiation of Social Interactions and Goal Meshing

As mentioned above, the results from Turner and Stammbach-Geering (12) prompted a more detailed investigation of social contact initiation by household cats and their humans. Turner's (13) team observed the mechanics of social interactions in 158 cat-owning households over three consecutive days, recording which partner, the cat or the person, tried to initiate the interaction (precisely defined), the reaction of the partner (accepting or declining), the duration of each interaction as well as total interaction time observed in that cat-human relationship. The goal of the project was to determine a potential measure of relationship success or quality. Firstly, Turner looked at the proportion of “intents” to interact that were successful - separately for the cat and the person (in this study, the woman of the household) - and attempted to correlate these values with total interaction time in the relationship over all cat-human dyads observed. There was no significant correlation for the cat data, but a significant negative one for the humans. The more successful the person was in initiating interactions, the *shorter* the total interaction time with the cat. This means that it is the cat that determines how long the interaction lasts. The next measure combined the data for the cats and humans into one number, namely, the proportion of all successful attempts to interact that were due to the cat. Over all person-cat pairs, this measure was indeed positively correlated with total interaction time in a relationship. That is the higher the proportion of all successful intents to interact that were due to the cat, the more time spent overall interacting in the relationship.

In Mertens' (34) observational study in other households, she found that the human partner was generally more active than the cat in distance regulation, especially in reducing distance between the two, but that single bouts of staying close to each other were longer when initiated by the cat. Further, Mertens reported a higher degree of reciprocity in distance regulation in cat-human dyads with adults than in those with children and juveniles, indicating a better “meshing” of close contact. “Goal meshing,” i.e., whether the goals of each partner are aligned with the ongoing goals of the other, is one important quality of any relationship (35).

Turner (13) continued the analysis of his data and calculated the proportion of “start interactions” (a defined and recorded element) due to the cat whenever the person had shown an intent to interact (also precisely defined), i.e., the individual cat's willingness to comply with the woman's “wish” to interact. Operationally, the “wish” to interact was defined for both the human and the cat as an approach to the partner and/or a directed vocalization. Also for each pair, whenever the cat had shown an intent to interact, he calculated the proportion of “starts” due to the woman, or, the woman's willingness to comply with the cat's “wish” to interact. These two values over all observed human-cat pairs were positively and significantly correlated. In other words, if the woman complies with the cat's wishes to interact, then the cat complies with the woman's wishes at other times; if the woman doesn't comply with the cat's wishes, then neither does the cat, with the woman's wishes. Therefore, a symmetry exists in the relationships at all levels of compliance, high to low, which might explain the popularity of cats, but also differences in the level of interactivity between relationships. In some relationships there is a high level of interactivity, in others, low, and the cat apparently accepts this, as indicated by staying on as the household pet (even when allowed outside) and lowering its own rate of initiation of interactions, when the owner shows less interactivity.

Wedl et al. (36) used a relatively new tool to analyze the structure of human-cat interactions observed in the home setting, namely Theme® (Noldus bv, The Netherlands). Strings of video recorded owner and cat behaviors were analyzed during four visits to each of 40 cat-owning households. The Theme® algorithm detects sets of events which follow each other non-randomly in the temporal sequence. Two actions that occur repeatedly and regularly in alternation form a basic “t-pattern.” Hierarchically structured t-patterns emerge via the detection of relationships of these previously detected patterns by repeated use of the algorithm scanning the strings of behaviors. Wedl and her co-workers found that owner and cat personality and gender and cat age of the partners (see below) had significant effects on t-patterning of dyadic behavior. In dyads with a female owner, the number of patterns per minute tended to be higher than in dyads with a male owner. Further, cat sex did not have any effect on the temporal patterning of dyadic behavior. These results are consistent with results found by Mertens [(34), see above] and Turner (1).

## Differences Related to Human Gender and Age

Mertens and Turner (37) reported differences found between the behavior of men, women, boys and girls in an experimental study of their colony cats. When the human volunteers were not allowed to interact in any way with the cats they were meeting for the first time in an encounter room (they had to look at an age-appropriate book during the first 5 min), the cats entering the room showed no preference for gender or age of the partner in their approach behavior. However, during the following 5 min when the persons were allowed to interact as they pleased with the cats and the authors recorded the human's behavior, the cats *reacted* to differences in behavior between men, women and children. Men tended to remain seated while women and girls moved down onto the floor, to the level of the cats. Children, especially the boys, tried to approach the cats immediately to which the cats usually reacted negatively by fleeing from them, even though they were all well-socialized. Women and girls spoke to the cats more often and the cats vocalized more often with them than with the men or boys.

These results were supported by later observations by Mertens (34) during 504 h in 51 cat-owning households with 162 persons and 72 cats. When at home, women spoke and interacted more with the cats than men did. Children were especially active with respect to motor activity, while adults spoke more often to the cats. She also found that interactions with women had a higher reciprocity and therefore probably both the person and the cat enjoyed high-quality relationships. In a more recent study, Wedl et al. (36) found that female owners entertained a more structured interaction with their cats than male owners and that extraverted owners have relatively varied interaction patterns with their animals. From a PCA analysis of answers to a questionnaire by Hungarian cat owners, Pongracz and Szapu (38) reported that women considered their cats to be more communicative and empathetic than men did and that emotional matching of the cat was more commonly reported by elderly owners than young owners.

Turner (39) compared the interactions of younger adults and elderly persons (65+) with their cats and found no difference in total interaction time between the two groups, but two differences in the structure of those interactions: Younger adults interacted significantly more often with their cats, but when older people interacted, they did so for significantly longer (Presumably the elderly waited until the cat came to them to interact, but this was not tested.). The younger owners also interacted more often from a distance and spoke more often to the cat than the elderly did.

All of the above findings have allowed recommendations to psychotherapists and pedagogues working with cats to help people in texts (40, 41) and courses in animal-assisted intervention, as well as to the general public to promote harmonious cat-human relationships.

## The Effects of Cats on Human Moods

Rieger and Turner (42) and Turner and Rieger (43) discovered that not only the mere presence of a cat in the household, but also interactions with the cat reduce measureable negative moods in the person, e.g., anxiety, depression, and introversion. The depressive owner initiates fewer interactions with the cat, but when the cat approaches that person, s/he accepts the intent of the cat to interact, which affects the human's mood. The cat also changes its behavior in response to depressiveness of the human when close to the person (but not at a distance), vocalizing more frequently with the person and head- and flank-rubbing more often on that person. More mood subscales in women than in men are affected by the cat, and they are more strongly affected than in men. Turner et al. (44) concluded that only the partner, but not the cat, enhances positive moods, while the cats alleviate negative moods. This effect was comparable to the effect of a human partner.

## Effects of Cat Breed and Age on Cat Behavior and Cat-human Interactions

Surprisingly, given the large number of popular cat breed books, there have been relatively few research studies of breed differences in behavior or behavior toward people. Turner (8, 39) reported on the only ethological study that compared the two oldest purebreds, Persian and Siamese cats, with non-pedigree cats and combined observational data with subjective trait ratings by the owners. He found few differences between the two breeds - reportedly at the extreme ends of cat personality - presumably due to convergent human selection, but those expected from the popular literature: The Persians were less active and less vocal than the Siamese, while the latter were more playful but demanding of their owners. Relative to the non-purebred cats, the purebreds were often closer to their owners and friendlier to strangers, which might be related to differences in handling (pampering) during upbringing or to artificial selection (genetic differences).

Hart and Hart (45) interviewed some 80 US-veterinarians in feline practices considered to be unbiased authorities on breed differences in cats. They ranked a random selection of five breeds and domestic short- and long-haired cats out of 15 cat breeds along 12 behavioral traits. Three traits had high predictive value to distinguish the breeds, seven traits with moderate and two traits with low predictive value. However, Turner (46) stated that confirmation of these subjective rankings is still needed from comparative ethological observations. The same criticism can be made of two more recent, but otherwise promising studies for future work, namely by Wilhelmy et al. (47) and Salonen et al. (48). Using a well-known questionnaire to generate standardized behavioral profiles, the former study found behavioral characteristics in purebred cats associated with breed, coat color and coat pattern. The latter study also gathered a large data set from a health and behavior questionnaire completed by owners and determined behavioral differences between 19 breeds and breed groups along 10 different behavior traits. A moderate level of heritability in three breeds for seven traits was found but the authors reported that substantial genetic variation exists within breed populations.

There are even fewer studies of the effect of cat age on cat-human interactions. Wedl et al. (36) employed the Theme® algorithm to their observational data and determined that the older the cat, the lower the dyadic event type complexity, meaning that the strings of cat behavior in interaction with their owners are shorter in old cats than young ones. This probably reflects decreased activity levels and playfulness with age in cats.

## Concluding Remarks

This mini-review has shown that we have discovered much about the mechanics of social interactions between cats and their owners, but that more remains to be discovered when researchers apply new techniques, e.g., phonetic analysis of cat vocalizations, or by applying the Theme® algorithm to analyze such interactions. More observational studies comparing the behavior of different cat breeds and animals of different coat characteristics would be welcomed to substantiate and compliment the owners' qualitative assessments of personality traits. Further, it is hoped that an ethically acceptable method to test the prediction of Turner's (1, 8) model on the effects of later positive and negative experiences with people on friendliness to people can be found.

## Author Contributions

The author confirms being the sole contributor of this work and has approved it for publication.

## Conflict of Interest

The author declares that the research was conducted in the absence of any commercial or financial relationships that could be construed as a potential conflict of interest.

## Footnotes

^1^www.humanesociety.org *Kitten behavior basics*.
